# Normobaric Hypoxia Exposure During Treadmill Aerobic Exercise After Stroke: A Safety and Feasibility Study

**DOI:** 10.3389/fphys.2021.702439

**Published:** 2021-08-16

**Authors:** Liam P. Kelly, Fabien Andre Basset, Jason McCarthy, Michelle Ploughman

**Affiliations:** ^1^Recovery and Performance Laboratory, Faculty of Medicine, Memorial University of Newfoundland, St. John’s, NL, Canada; ^2^School of Human Kinetics and Recreation, Memorial University of Newfoundland, St. John’s, NL, Canada

**Keywords:** stroke, rehabilitation, aerobic exercise, normobaric hypoxia, altitude training, secondary prevention, maximum oxygen uptake

## Abstract

**Objective:**

To evaluate the safety and feasibility of performing treadmill aerobic exercise in moderate normobaric hypoxia among chronic hemiparetic stroke survivors.

**Design:**

Observational study using convenience sampling.

**Setting:**

Research laboratory in a tertiary rehabilitation hospital.

**Participants:**

Chronic hemiparetic stroke survivors who could walk at least 10-m with or without assistance and had no absolute contraindications to exercise testing.

**Intervention:**

Participants (three male and four female) were asked to complete three normobaric hypoxia exposure protocols within a single session. First, they were passively exposed to normobaric hypoxia through gradual reductions in the fraction of inspired oxygen (F_I_O_2_ = 20.9, 17.0, and 15.0%) while seated (5-min at each level of F_I_O_2_). Participants were then exposed to the same reductions in F_I_O_2_ during constant-load exercise performed on a treadmill at 40% of heart rate reserve. Finally, participants completed 20-min of exercise while intermittently exposed to moderate normobaric hypoxia (5 × 2-min at F_I_O_2_ = 15.0%) interspaced with 2-min normoxia intervals (F_I_O_2_ = 20.9%).

**Outcome Measures:**

The primary outcome was occurrence of adverse events, which included standardized criteria for terminating exercise testing, blood oxygen saturation (S*p*O_2_) <80%, or acute mountain sickness score >2. The increased cardiovascular strain imposed by normobaric hypoxia exposure at rest and during exercise was evaluated by changes in S*p*O_2_, heart rate (HR), blood pressure, and rating of perceived exertion (RPE).

**Results:**

One participant reported mild symptoms of nausea during exercise in normobaric hypoxia and discontinued participation. No other adverse events were recorded. Intermittent normobaric hypoxia exposure was associated with reduced S*p*O_2_ (MD = −7.4%, CI: −9.8 to −5.0) and increased HR (MD = 8.2, CI: 4.6 to 11.7) compared to intervals while breathing typical room air throughout the 20-min constant-load exercise period. The increase in HR was associated with a 10% increase in relative effort. However, reducing F_I_O_2_ had little effect on blood pressure and RPE measurements.

**Conclusion:**

Moderate normobaric hypoxia appeared to be a safe and feasible method to increase the cardiovascular strain of submaximal exercise in chronic hemiparetic stroke survivors. Future studies evaluating the effects of pairing normobaric hypoxia exposure with existing therapies on secondary prevention and functional recovery are warranted.

## Introduction

Poor cardiorespiratory fitness is characteristic of individuals experiencing the effects of stroke. Measurements taken during the first 3 months of recovery demonstrate that maximum oxygen uptake values range from 10 to 18 mL min^–1^ kg^–1^ (Mackay-Lyons and Makrides, 2004; Tang et al., 2006; Mackay-Lyons et al., 2013). Given the inverse association between cardiorespiratory fitness and future risk of stroke (Pandey et al., 2016) it is perhaps not surprising that these values correspond to the “very poor” category based on normative data (Riebe et al., 2018). However, it is striking that fitness levels among stroke survivors are less than 60% of that reported for age and sex-matched coronary heart disease patients upon completion of cardiac rehabilitation (Banks et al., 2019). Such differences in fitness could be explained by the fact that people with cerebrovascular disease are offered lower-intensity task-oriented rehabilitation compared to people having cardiovascular disease. (Hebert et al., 2016). Although relevant to overcome functional limitations, task-oriented therapies offered during stroke rehabilitation provide insufficient cardiovascular stress (MacKay-Lyons and Makrides, 2002; Barrett et al., 2018).

Important advances are taking place in the field of stroke rehabilitation to enhance the provision of aerobic training (Biasin et al., 2014); specifically transitioning eligible patients with stroke to cardiac rehabilitation (Marzolini, 2018). Feasibility and preliminary effectiveness of comprehensive cardiac rehabilitation programs has been confirmed among individuals who have experienced a transient ischemic attack or non-disabling minor stroke (Prior et al., 2011). The challenge, however, is adapting such programs for individuals with more severe stroke-related impairments (Tang et al., 2009). Indeed, stroke survivors with mild-to-moderate levels of motor impairments can meet the minimal aerobic exercise targets (Marzolini et al., 2012) and achieve statistically significant increases in pre-to-post measures of cardiorespiratory fitness upon completion of adapted cardiac rehabilitation programs (Lennon et al., 2008; Tang et al., 2010; Marzolini et al., 2012, 2014). However, cardiorespiratory fitness remains in the “very poor” category upon completion of adapted cardiac rehabilitation and the training-induced improvements in cardiometabolic risk factors have been inconsistent (Lennon et al., 2008; Tang et al., 2010; Marzolini et al., 2014). Accordingly, new approaches are needed to enhance the effects of physical exercise interventions currently offered to stroke survivors.

Normobaric hypoxia conditioning has the potential to enhance the effects of exercise in stroke survivors. Normobaric hypoxia exposure involves reductions in the fraction of inspired oxygen (F_I_O_2_) from values recorded near sea-level (F_I_O_2_ = 20.9%) to values that simulate the moderate levels of hypoxia experienced at elevations between 2,000 and 3,000 m above sea-level (F_I_O_2_ = 17.0 to 15.0%) (Bartsch and Saltin, 2008). During normobaric hypoxia conditioning studies, participants perform submaximal exercise while breathing the normobaric hypoxia gas mixture (Klug et al., 2018). Since maximum aerobic workloads decrease at a rate of about 7% for every 1,000 m increase in altitude (≅ 2% decrease in F_I_O_2_) (Fulco et al., 1998), the absolute workloads performed in normobaric hypoxia must be reduced to be equivalent to the relative exercise intensity experienced under typical sea-level conditions (Bartsch and Saltin, 2008). In other words, training in normobaric hypoxia is a method to increase the cardiometabolic stress of submaximal exercise without an increase in biomechanical strain (Pramsohler et al., 2017). Interestingly, among overweight/obese participants, exercise completed in normobaric hypoxia conditions resulted in similar (Klug et al., 2018) or even enhanced (Netzer et al., 2008; Wiesner et al., 2010) benefits on cardiometabolic outcomes compared to aerobic workloads matched for relative intensity at sea-level. Clinically, the approximately 15% increase in relative effort during submaximal exercise performed in moderate normobaric hypoxia (Fulco et al., 1998) may provide a unique solution for stroke survivors with diminished ability to perform external work due to hemiparesis. Although preliminary evidence in other clinical populations suggests a synergistic effect of pairing exercise with normobaric hypoxia exposure on metabolic (Mackenzie et al., 2011; Mahler et al., 2018) and functional outcomes (Navarrete-Opazo et al., 2017b), more work is needed to translate these findings to stroke survivors. Accordingly, the objective of the current preliminary study was to determine if exposing chronic hemiparetic stroke survivors to moderate levels of normobaric hypoxia during treadmill aerobic exercise was safe and feasible.

## Materials and Methods

### Participants

Participants were recruited from a registry of chronic stroke survivors who completed inpatient/outpatient rehabilitation services and recently participated in a rehabilitation trial at our laboratory (NC1674790). Participants had to meet the following inclusion criteria to be considered for the current study: (1) age ≥ 18 years, (2) ischemic or hemorrhagic stroke > 6 months before study enrollment, (3) ability to perform 2-step instruction, (4) and ambulatory with or without aid > 10 m. Participants were excluded if they presented with any absolute contraindications for graded exercise testing as described elsewhere (Riebe et al., 2018) or reported a change in physical activity levels (increase or decrease) since their last graded exercise test. The study protocol was approved by the regional Health Research Ethics Board (2018.013) in accordance with the TCPS (II) guidelines (Canadian Institutes of Health Research et al., 2018) and the declaration of Helsinki.

### Physical Profile

Data collected during the follow-up period of the previous trial, including assessments of stroke severity (National Institute of Health Stroke Scale, and Chedoke McMaster stage of recovery for leg and foot) (Goldstein et al., 1989; Gowland et al., 1993), walking performance, and symptom-limited graded exercise testing, were used in the current study to describe participants’ physical profile. As described elsewhere (Ploughman et al., 2019), self-selected walking speed was assessed on an instrumented gait analysis walkway (Zeno Walkway, Protokinetics LLC, PA, United States) and the average of two trials were recorded. Symptom-limited graded exercise tests were performed on either a motorized treadmill (SportsArt T652M, WA United States) with harness support (< 10% of body weight, PneuWeight, Pneumex, ID, United States) or a total-body recumbent stepper (T4r, Nustep, LLC, MI, United States) as described previously (Kelly et al., 2017). Maximum heart rate (HR), maximum oxygen uptake (V̇O_2max_), and the HR corresponding to 40% of V̇O_2max_ were recorded for use in the current study.

### Experimental Design

**FIGURE 1:**
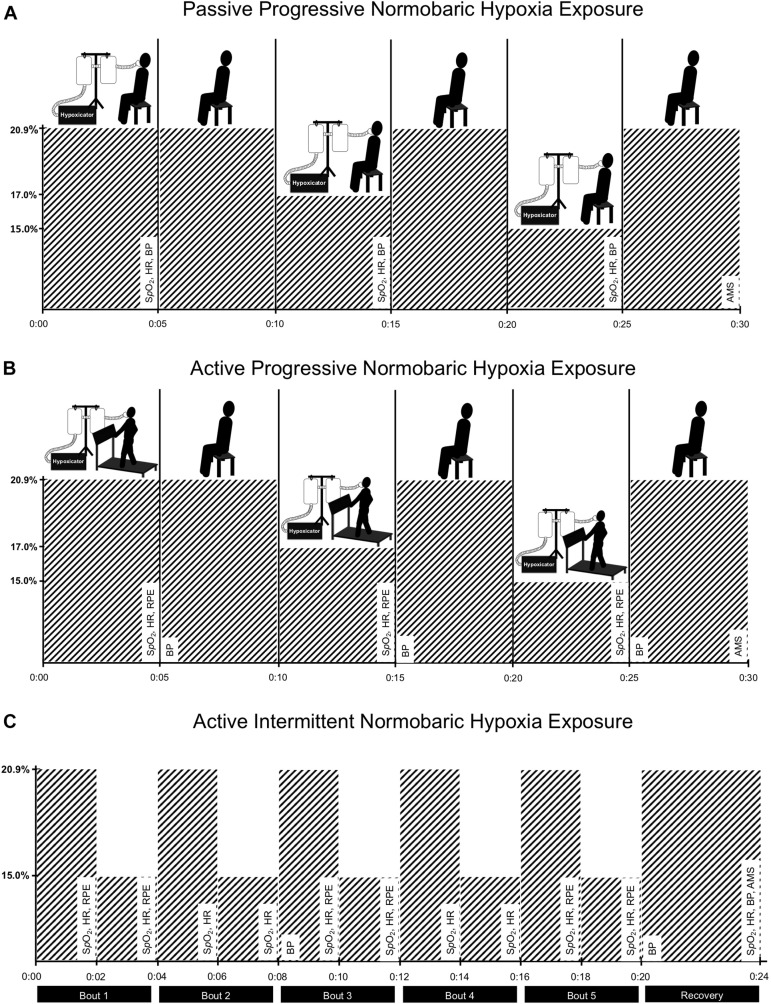
Experimental design. To evaluate the safety and feasibility of moderate normobaric hypoxia exposure, participants were exposed to gradual reductions in the fraction of inspired oxygen (F_I_O_2_) during sitting [panel **(A)**] and during constant-load treadmill walking [panel **(B)**]. Then, participants completed 20-min of constant-load treadmill walking while intermittently exposed to normobaric hypoxia (5 × 2-min at F_I_O_2_ = 15.0%) interspaced with intervals of typical room air (normoxia; 5 × 2-min at F_I_O_2_ = 20.9%) [panel **(C)**]. Each pair of normobaric hypoxia and normoxia intervals were defined as a bout (5-bouts in total). Measures of blood oxygen saturation (S*p*O_2_), heart rate (HR), blood pressure (BP), rating of perceived exertion (RPE), and symptoms associated with more prolong altitude exposure using the Lake Louise Acute Mountain Sickness Scale (AMS) were monitored throughout the three exposure protocols and recorded at defined intervals as indicated in the figure. A hypoxicator using nitrogen filtration technique was used to manipulate fraction of inspired oxygen in two Douglas bags. Participants were interfaced with the bags using corrugated tubing, non-breathable valve, and an oro-nasal facemask.

Given the preliminary objectives of this observational study, the aim was to recruit 8 participants (4 females). Study participants were asked to complete three different normobaric hypoxia exposure protocols within a single session. As illustrated in Figure 1, F_I_O_2_ was manipulated using a hypoxicator (GO2Altitude, Biomedtech Ltd., VIC, Australia) that continuously pumped air (120 L min^–1^) into two 120-liter Douglas bags. Gas concentration within the Douglas bags was monitored using an oxygen sensor (Rapidox O_2_, Sensotec Ltd., Cambridge, United Kingdom), ensuring that the target F_I_O_2_ was maintained within ± 0.2%. Participants were interfaced with the hypoxicator using a two-way non-rebreathing valve (2700, Hans Rudolph, Kansas, United States) connected to an oro-nasal facemask (series 8900, Hans Rudolph, Kansas, United States), and tubing. Blood pressure (BP; systolic/diastolic), HR (using 12-lead electrocardiography; CardoCard, Nasiff Associates Inc., NY, United States), oxygen saturation (S*_*p*_*O_2_; Massimo SET, CA, United States), and rating of perceived exertion (RPE) (Borg, 1982) were monitored throughout the three normobaric hypoxia exposure protocols, which are described below. Also, potential symptoms (headache, nausea, and dizziness/light-headedness) typically associated with more prolonged altitude exposure (i.e., > 6 h.) were monitored at the end of each exposure protocol and again the next morning over the telephone using the Lake Louise Acute Mountain Sickness Scale (Roach et al., 2018).

#### Passive Progressive Normobaric Hypoxia Exposure Protocol

During the first exposure protocol, participants were passively exposed to gradual reductions in F_I_O_2_ during seated rest. As illustrated in Figure 1A, participants first breathed a gas mixture consistent with typical room air (i.e., normoxia; F_I_O_2_ = 20.9%) for 5-min and then F_I_O_2_ was reduced to 17.0% for an additional 5-min and finally reduced to 15.0% for the last 5-min of the passive progressive normobaric hypoxia exposure protocol. Measures of HR, S*p*O_2_, and BP were recorded during the last 30-s of exposure at the three levels of F_I_O_2_ studied. The facemask was removed between each 5-min exposure period to allow time (5-to 10-min) for the adjustment of F_I_O_2_ within the Douglas bags.

If no adverse events were recorded during passive progressive normobaric hypoxia exposure, participants moved on to the active phases of the study where normobaric hypoxia exposure was paired with constant-load exercise performed on the treadmill with bodyweight supported (∼10%) at self-selected walking speed. To determine self-selected walking speed, participants were first familiarized with the treadmill and then asked to choose a speed that they could comfortably maintain for 20 min or more. Treadmill incline was then adjusted to achieve a target HR corresponding to 40% of participants heart rate reserve while breathing normoxia room air.

#### Active Progressive Normobaric Hypoxia Exposure Protocol

During the second exposure protocol, participants were exposed to gradual reductions in F_I_O_2_ while walking on the treadmill at the predetermined treadmill speed and incline. As illustrated in Figure 1B, the same protocol described above was used to progressively increase the level of normobaric hypoxia exposure during treadmill walking in 5-min epochs. Measures of HR, S*p*O_2_, BP, and RPE were recorded during the last 30-s of exposure at the three levels of F_I_O_2_ studied. Again, if no adverse events were recorded, participants moved on to the final exposure protocol.

#### Active Intermittent Normobaric Hypoxia Exposure Protocol

During the third exposure protocol, participants walked on the treadmill at the predetermined speed and incline for 20-min while intermittently exposed to 5 × 2-min intervals at F_I_O_2_ = 15% interspaced with 2-min intervals at F_I_O_2_ = 20.9% (see Figure 1C). During active intermittent normobaric hypoxia exposure, gas concentration remained constant within the Douglas bags (i.e., 15.0 ± 0.2%) and a valve was used to switch between room air and moderate normobaric hypoxia every 2-min. Measures of HR and S*p*O_2_ were recorded during the last 30-s of each 2-min interval.

### Outcome Measures

The primary objective of the current study was to determine if exposing chronic hemiparetic stroke survivors to moderate levels of normobaric hypoxia during treadmill aerobic exercise was safe and feasible. Accordingly, having to stop the protocol due to occurrence of adverse events and circumstances for termination were of primary importance. The normobaric hypoxia exposure periods were terminated according to accepted indications for stopping an exercise test (Riebe et al., 2018). Specifically, the exercise was halted if there was a decrease in systolic blood pressure (< 10 mm Hg) below baseline values or a hypertensive response (systolic > 250 mm Hg or diastolic > 115 mm Hg), a significant change in the electrocardiography tracing (e.g., ST elevation/depression, ventricular tachycardia, PVC’s, etc.), onset of angina like symptoms, shortness of breath, signs of severe fatigue, or participant requested to stop. Given the anticipated effects of normobaric hypoxia exposure on S*_*p*_*O_2_, especially among individuals treated with beta blockers (Bilo et al., 2011), a cutoff score of 80% was set for exercise termination. Participants could only proceed from one exposure protocol to the next if there was absence of termination indicators. In terms of feasibility, participants’ ability to sustain constant workloads under moderate normobaric hypoxia was evaluated along with changes in cardiovascular strain.

### Statistical Analysis

Effects of normobaric hypoxia exposure on HR, S*p*O_2_, and BP were evaluated at the levels of F_I_O_2_ studied during seated and treadmill walking activities. The Shapiro–Wilk test was first performed to determine if the data come from an approximately normal distribution and log transformed if required. Repeated measures one-way ANOVA was used to test for differences at the three levels of F_I_O_2_ during the passive progressive normobaric hypoxia and active progressive normobaric hypoxia exposure protocols. Repeated measures two-way ANOVA was used to test for differences in HR and S*p*O_2_ response between the 5-intervals of normoxia (F_I_O_2_ = 20.9%) and normobaric hypoxia (F_I_O_2_ = 15.0%) during the active intermittent exposure protocol. Changes in RPE were recorded during the active treatments only and evaluated using the Friedman test and Wilcoxon matched pairs signed rank test during the progressive and intermittent protocols, respectively. Ratio data are reported as mean difference (MD) and 95% confidence interval (CI), unless otherwise indicated. Ordinal data are reported as the median and range. Statistical significance was set at *p* < 0.05. All statistic tests and figures were prepared using Prism 8 for MacOS (GraphPad Software, CA, United States).

## Results

### Participants

Thirteen potential participants were contacted; four declined to participate, one was excluded during the medical examination due to uncontrolled diabetes and peripheral neuropathy, and one participant dropped out prior to the intervention. Characteristics of the remaining seven participants are displayed in Table 1. Participants were on average 5.7 years (SD 0.8) since their first disabling stroke, most often due to ischemia. The cohort had mild to moderate stroke severity (National Institute of Health Stroke Scale; median = 2, range = 0–9) (Kwakkel et al., 2010) and incomplete sensorimotor recovery of the lower-limb according to the Chedoke-McMaster Stroke Assessment (Gowland et al., 1993) (leg + foot/14; median = 9.0, range = 4–13). Self-selected walking speed was below the 80 cm s^–1^ threshold set for community ambulation in 3/7 subjects (Perry et al., 1995; Duncan et al., 2011). All participants were receiving treatment for hypertension and most had at least one metabolic comorbidity. Average BMI was 28.5 kg m^–2^ (SD 6.9); corresponding to the overweight category (Riebe et al., 2018). Fitness levels were in the “very poor” to “poor” range [18.7 ml min^–1^ kg^–1^ (SD 5.0)] compared to age and sex matched normative data (Riebe et al., 2018). Maximum HR achieved during the prior symptom-limited graded exercise test was 149 bpm (SD 28), which was within 10% of age predicted values.

**TABLE 1:** Participant characteristics.

Participant #	1	2	3	4	5	6	7
Age (years)	83	63	57	65	53	73	48
Sex (Male/Female)	F	F	M	F	F	M	M
Weight (kg)	60.0	65.5	71.9	60.0	85.3	90.8	64.9
Stroke type	Ischemic	Hemorrhagic	Ischemic	Ischemic	Hemorrhagic	Ischemic	Ischemic
Time since stroke (years)	5.6	4.9	5.8	6.7	6.6	4.8	5.1
Stroke severity (NIHSS^1^; low 0 to 42 high)	0	2	9	2	5	1	7
Chedoke-McMaster Leg/Foot Impairment (low 7 to high 1)	6/6	5/4	5/3	5/5	5/4	7/6	3/1
Resting Heart Rate	52	55	63	71	88	72	88
Resting BP (mmHg)	130/80	110/60	142/90	110/70	140/80	134/66	90/60
Walking speed (cm s^–1^)	66.8	103.7	46.7	75.4	83.5	116.3	89.9
V̇O_2max_ (mL min^–1^ kg^–1^)	14.2	25.1	15.0	16.6	14.0	20.3	25.7
Cardiorespiratory fitness category^2^	VP	P	VP	VP	VP	VP	VP
Maximum HR^3^	126	137	105	153	171	168	185
Age predicted Maximum HR	151	120	124	163	171	158	175
Dyslipidemia	Yes	No	Yes	Yes	No	Yes	Yes
Diabetes	No	No	No	Yes	No	Yes	Yes
Hypertension	Yes	Yes	Yes	Yes	Yes	Yes	Yes
Beta blocker (yes/no)	No	Yes	Yes	No	No	No	No
Osteoporosis/Osteopenia	Yes	Yes	Yes	Yes	No	No	No

*^1^NIHSS, National Institute of Health Stroke Scale. ^2^based on age and sex matched normative data published by the American College of Sports Medicine (Riebe et al., 2018); VP, very poor (< 20th percentile); P, poor (<40th percentile). ^3^maximum value recorded during a symptom-limited graded exercise test.*

All participants were able to complete the passive progressive normobaric hypoxia exposure period and no adverse events were reported. A gradual reduction in S*p*O_2_ was observed at rest during exposure to moderate normobaric hypoxia compared to the normoxia condition. The mean difference and 95% confidence interval (CI) were 2.1% (0.9 to 3.4) and 4.6% (1.5 to 7.6) at F_I_O_2_ equal to 17.0 and 15.0%, respectively. A corresponding increase in resting HR was observed with reductions in F_I_O_2_ (MD = 4.1 bpm, CI: 1.4 to 6.9 and MD = 4.6 bpm CI: −0.1 to 9.3, respectively). Systolic and diastolic blood pressures remained relatively stable throughout the passive progressive normobaric hypoxia exposure protocol.

**FIGURE 2:**
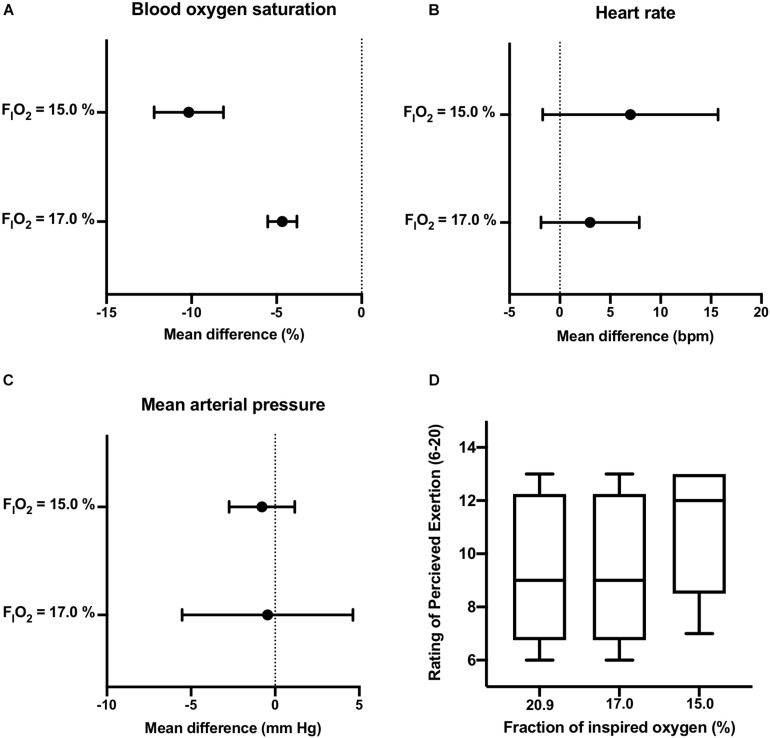
Active Progressive Normobaric Hypoxia Exposure. Effects of reducing F_I_O_2_ on blood oxygen saturation [panel **(A)**], heart rate [panel **(B)**], mean arterial blood pressure [panel **(C)**], and rating of perceived exertion [panel **(D)**] during constant workload exercise. Data reported as mean difference and 95% confidence interval for the difference between normoxia (F_I_O_2_ = 20.9%) and the two levels of normobaric hypoxia (F_I_O_2_ = 17.0, and 15.0%). The box and whisker plots [panel **(D)**] report the median along with the 25th and 75th percentiles and range (min to max) at the three levels of F_I_O_2_ studied.

Participants were able to maintain constant-load treadmill walking at workloads corresponding to 40% of HRR in normoxia (see Supplementary Table 1). One participant reported mild symptoms of nausea after initiation of exercise at F_I_O_2_ = 17.0% and requested to stop. No adverse events were reported in the remaining participants. As displayed in Figure 2, dose response changes in S*p*O_2_ were observed during exercise performed under normobaric hypoxia. Importantly, reductions in S*p*O_2_ were above the cutoff score. Corresponding increases in HR were observed at the two levels of F_I_O_2_ studied. The increased cardiovascular strain imposed by moderate normobaric hypoxia exposure did not alter blood pressure responses to constant-workload exercise. Also, reducing F_I_O_2_ had little effect on participants RPE during exercise.

**FIGURE 3:**
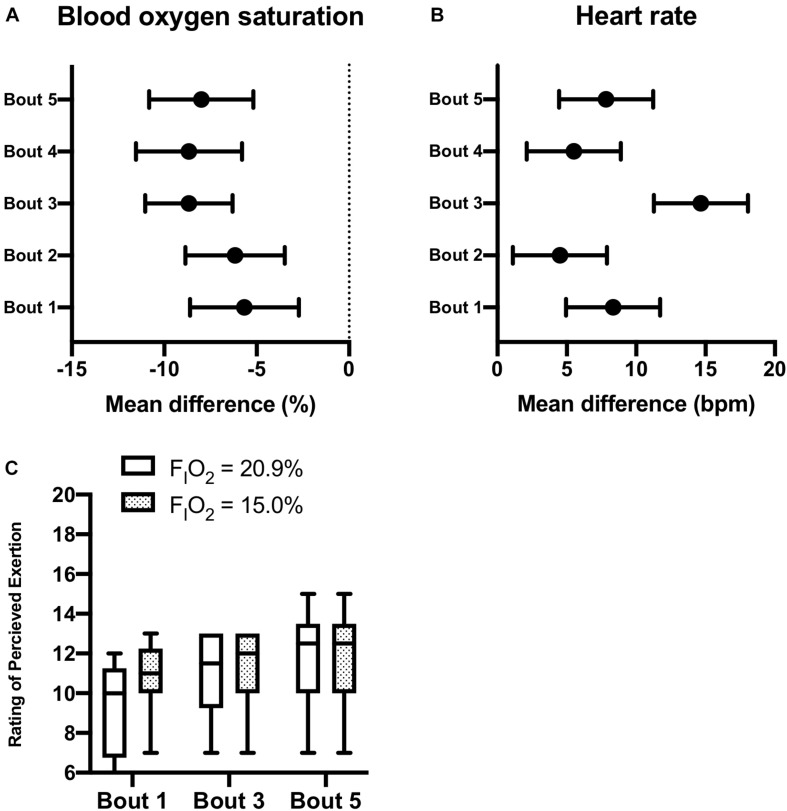
Active Intermittent Normobaric Hypoxia Exposure. Effects of intermittent normobaric hypoxia exposure (F_I_O_2_ = 15.0%) on blood oxygen saturation [panel **(A)**], heart rate [panel **(B)**], and rating of perceived exertion [panel **(C)**] during constant workload exercise. Data reported as mean difference and 95% confidence interval for the difference between 2-min intervals of normoxia (F_I_O_2_ = 20.9%) and normobaric hypoxia (F_I_O_2_ = 20.9%). Each pair of normobaric hypoxia and normoxia intervals were defined as a bout (5-bouts in total). The box and whisker plots [panel **(C)**] report the median along with the 25th and 75th percentiles and range (min to max) for rating of perceived exertion during constant workload exercise at the two levels of F_I_O_2_ studied during bouts 1, 3, and 5.

Participants (*n* = 6) were able to maintain 20-min of constant-load treadmill walking during intermittent (5-intervals of 2-min) exposure to moderate normobaric hypoxia (F_I_O_2_ = 15.0%) interspaced with 5-intervals of 2-min at F_I_O_2_ = 20.9%. No adverse events were reported during AINH. As described in Figure 3, pairs of normobaric hypoxia and normoxia exposure were defined as bouts (i.e., 5-bouts in total). A significant group × time effect was observed for reductions in S*p*O_2_ during normobaric hypoxia exposure compared to normoxia across the 5-bouts (*p* = 0.017). Correspondingly, group × time effects were observed for increases in HR during normobaric hypoxia exposure compared to normoxia across the 5-bouts (*p* = 0.003). Although two participants reported a slightly higher RPE during the first interval in normobaric hypoxia, scores were essentially the same by the final bout (see Figure 3). No symptoms of headache, nausea, or dizziness/light-headedness were reported the next morning over the telephone.

## Discussion

The current observational study was undertaken to examine the preliminary safety and feasibility of performing treadmill aerobic exercise under conditions of moderate normobaric hypoxia among chronic hemiparetic stroke survivors. Other than one participant reporting mild symptoms of nausea upon reducing F_I_O_2_ during exercise, no adverse events were observed during normobaric hypoxia exposure or the morning after. As anticipated, the addition of moderate normobaric hypoxia to constant workload exercise was associated with reductions in S*p*O_2_ and corresponding increases in HR. However, the increased cardiovascular strain had little effect on BP measurements and participants did not perceive the efforts as more demanding, as indicated by RPE scores. The current study provides initial evidence that supports the use of normobaric hypoxia exposure to increase the cardiovascular demands of submaximal exercise in chronic stroke survivors with walking impairments.

Several research reports have evaluated the safety of exposure to environments with reduced oxygen availability, such as terrestrial high altitudes (i.e., hypobaric hypoxia), in older adults with preexisting cardiovascular conditions (Bartsch and Gibbs, 2007; Levine, 2015; Parati et al., 2018). It is generally accepted that individuals with asymptomatic coronary heart disease who have a sufficiently high exercise capacity (i.e., > 6 metabolic equivalents or 21 ml min^–1^ kg^–1^) are at minimal increased risk during high altitude trekking (Bartsch and Gibbs, 2007). However, as reflected in the current data, the majority of stroke survivors have cardiorespiratory fitness levels below this threshold (Smith et al., 2012; Saunders et al., 2020). Also, hemiparetic gait is associated with a 1.5 to twofold increased energy demand (Kramer et al., 2016). The net effect of low cardiorespiratory fitness and increased energy cost of ambulatory activities is a diminished capacity to accommodate changes in aerobic demand. With the goal of developing normobaric hypoxia conditioning protocols appropriate for stroke rehabilitation, we investigated whether chronic hemiparetic stroke survivors could tolerate treadmill walking while progressively and intermittently exposed to reductions in F_I_O_2_. Arterial oxygen saturation, electrocardiography, blood pressure, and rating of perceived exertion were recorded during constant-load exercise to monitor for signs of excessive cardiovascular stress. Unremarkable changes in blood pressure and electrocardiography tracings were recorded during normobaric hypoxia exposure. These outcomes are consistent with data collected in a relatively large cohort (*N* = 97) of older adults upon initial ascent to high altitude (Erdmann et al., 1998). As anticipated, normobaric hypoxia exposure reduced S*p*O_2_ both at rest and during constant workload exercise. However, S*p*O_2_ values remained above the cutoff score in all participants, including those prescribed beta blockers, which has previously been identified as a potential risk among individuals receiving antihypertensive treatment (Bilo et al., 2011). Mild symptoms of nausea were reported by one participant, which dissipated shortly after stopping the exercise protocol. As reported in the Supplementary Table 1, this participant (#5) selected a treadmill speed above the target workload and it is unclear if the symptoms of nausea were a result of normobaric hypoxia itself or the high-intensity nature of such workloads when paired with normobaric hypoxia. No symptoms of headache, nausea, or dizziness/light-headedness were reported in the remaining participants. Collectively, findings from this preliminary study support that performing submaximal exercise under conditions of moderate normobaric hypoxia provides no additional risk beyond that of exercise in typical environments among chronic hemiparetic stroke survivors.

With respect to feasibility, participants were able to sustain 20-min of constant workload exercise while intermittently exposed to moderate normobaric hypoxia. Although the treadmill speed and incline were maintained throughout AINH, average HR was 7.8 bpm higher during the five 2-min intervals of normobaric hypoxia exposure. This corresponded to a 10% increase in relative effort based on participants’ HRR. Although lower than the anticipated 15% increase in relative effort for the level of F_I_O_2_ studied (Fulco et al., 1998), the change in relative effort is likely underestimated given that HRR is diminished under conditions of reduced oxygen availability (Bartsch and Gibbs, 2007). It is equally important to point out that HR and S*p*O_2_ began to recover between intervals, which suggest that participants were able accommodate to the increased cardiovascular stain of moderate normobaric hypoxia exposure during submaximal exercise. Furthermore, participants did not perceive the workloads as more demanding when performed at reduced F_I_O_2_. The lack of effect on RPE is consistent with the results of a previous placebo-controlled trial that reported participants were unable to accurately predict whether or not they performed submaximal exercise in normobaric hypoxia or under typical sea level conditions (Netzer et al., 2008). Accordingly, normobaric hypoxia exposure appears to be a feasible method of increasing the cardiovascular strain of submaximal exercise in chronic hemiparetic stroke survivors.

The focus of the current study was on using normobaric hypoxia exposure to increase the cardiovascular strain of submaximal exercise among chronic hemiparetic stroke survivors. However, normobaric hypoxia exposure may enhance the effects of submaximal exercise independent of changes in relative exercise intensity. For instance, in a recent randomized trial among overweight/obese females, metabolic benefits were enhanced when normobaric hypoxia was combined with 12-weeks of high-intensity interval training (Camacho-Cardenosa et al., 2018). This is consistent with normobaric hypoxia conditioning studies that report similar (Klug et al., 2018) or enhanced (Netzer et al., 2008; Wiesner et al., 2010) effects on cardiometabolic outcomes at lower absolute workloads. Furthermore, intermittent normobaric hypoxia exposure enhanced the effects of locomotor training on walking speed, endurance, and dynamic balance among individuals with incomplete spinal cord injury (Navarrete-Opazo et al., 2017b). The latter studies incorporated more severe levels of normobaric hypoxia (F_I_O_2_ = 9.0%) immediately prior to bodyweight supported treadmill training, which suggests a priming effect of normobaric hypoxia exposure. Although mechanisms underlying the synergistic effects of pairing normobaric hypoxia exposure with exercise are yet to be elucidated, both acute physiological responses and more prolonged acclamatory processes are likely involved (Hochachka et al., 1999). For example, during constant workload exercise performed in hypoxia there is an increased mobilization of carbohydrate and lipid energy substrates compared to exercise performed in normoxia at the same absolute and relative workloads (Peronnet et al., 2006). Despite the fact that increased mobilization of both energy substrates is characteristic of hypoxia exposure, substrate partitioning is shifted toward carbohydrate energy sources during exercise in hypoxia and lipid oxidation is enhanced during the post exercise recovery period under normoxic conditions (Kelly and Basset, 2017). Such metabolic perturbations during and in-between individual bouts of exercise may help to explain the similar or enhanced effects of normobaric hypoxia exposure on adiposity and metabolic profile among sedentary overweight adults at a lower total energy expenditure of exercise (Haufe et al., 2008; Netzer et al., 2008; Wiesner et al., 2010). Further, hypoxia-induced increases in the expression of transcriptional factors including hypoxia-inducible factor-1alpha (HIF-1 alpha) and proliferator-activated receptor-gamma coactivator-1 alpha (PGC-1 alpha) have been linked to improvements in insulin sensitivity and skeletal muscle lipid metabolism after normobaric hypoxia conditioning, respectively (Haufe et al., 2008; Wiesner et al., 2010). Normobaric hypoxia exposure also increases expression of brain derived neurotrophic factor and its receptor tyrosine kinase receptor-B (TrkB) (Dale et al., 2014), which is reasoned to explain the beneficial effects of intermittent hypoxia on ambulatory function among individuals with incomplete spinal cord injury (Navarrete-Opazo et al., 2017a). Given the benefits reported in other clinical populations and the multiple potential mechanisms underlying acute and more prolonged physiological responses, studies investigating the dose response effects of pairing normobaric hypoxia with task-oriented activity after stroke are warranted.

There are several methodological considerations that must be discussed. Firstly, the current study was conducted in a small convenience sample of chronic stroke survivors. This cohort was chosen because of their previous experience with aerobic exercise and the fact that they were able to achieve moderate intensity workloads without adverse events in a previous trial. In order to prepare for future studies, we needed to demonstrate that the additional risk associated with normobaric hypoxia exposure was proportional to its potential benefits. Secondly, we exposed participants to a narrow range of normobaric hypoxia both at rest and during a single constant workload. This dose of normobaric hypoxia exposure was chosen because of previous intervention studies conducted in sedentary populations that demonstrated favorable outcomes when submaximal aerobic exercise was paired with similar reductions in F_I_O_2_ (Haufe et al., 2008; Wiesner et al., 2010; Camacho-Cardenosa et al., 2018; Fernandez Menendez et al., 2018). Given the range of HR and S*p*O_2_ responses observed and the fact that perception of effort was not influenced by normobaric hypoxia exposure, this cohort likely could have tolerated even further decreases in F_I_O_2_ and/or increases in absolute workload. Also, decreases in S*p*O_2_ during exercise performed in normobaric hypoxia were highly variable between participants and individual adjustments in F_I_O_2_ may be required to ensure similar levels of cardiovascular and metabolic stress between participants (Lee and Thake, 2018).

## Conclusion

The current preliminary study provides initial evidence that moderate normobaric hypoxia exposure is a safe and feasible method to increase the cardiovascular strain of submaximal exercise after stroke. Scientific evidence collected over the last three decades indicates that stroke survivors can respond positively to aerobic exercise training (Saunders et al., 2020). However, the doses of aerobic exercise studied thus far are insufficient to enhance cardiorespiratory fitness to more normal and low risk levels. Stroke-related impairments and comorbid health conditions are significant barriers to sustaining workloads associated with moderate intensity aerobic exercise in this population (Biasin et al., 2014; Moncion et al., 2020). Accordingly, normobaric hypoxia exposure may be an effective strategy to enhance the effects of aerobic exercise training on cardiorespiratory fitness, secondary prevention, and functional recovery after stroke. Future studies are needed to confirm the results of the current study and to determine the most appropriate protocols for pairing normobaric hypoxia exposure with physical exercise in large representative samples of stroke survivors at different stages of recovery.

## Data Availability Statement

The raw data supporting the conclusions of this article will be made available by the authors, without undue reservation.

## Ethics Statement

The studies involving human participants were reviewed and approved by Newfoundland and Labrador Health Research Ethics Board. The patients/participants provided their written informed consent to participate in this study.

## Author Contributions

LK, FB, JM, and MP contributed to the conception and design of the study. LK and JM collected the data. LK performed the statistical analysis and wrote the first draft of the manuscript. All authors contributed to manuscript revision, read, and approved the submitted version.

## Conflict of Interest

The authors declare that the research was conducted in the absence of any commercial or financial relationships that could be construed as a potential conflict of interest.

## Publisher’s Note

All claims expressed in this article are solely those of the authors and do not necessarily represent those of their affiliated organizations, or those of the publisher, the editors and the reviewers. Any product that may be evaluated in this article, or claim that may be made by its manufacturer, is not guaranteed or endorsed by the publisher.
