# Supplementary material for: Normobaric Hypoxia Exposure During Treadmill Aerobic Exercise After Stroke: A Safety and Feasibility Study
Source: Front Physiol. 2021 Aug 16;12:702439. doi: 10.3389/fphys.2021.702439 (PMC8415265; doi:10.3389/fphys.2021.702439)
Supplement: Supplementary file 1 [file Table_1.DOCX]

**Supplementary Table 1: Constant workload exercise and heart rate responses at the three levels of fraction of inspired oxygen studied during active progressive normobaric hypoxia exposure**

| **Participant #** | **1** | **2** | **3** | **4** | **5** | **7** | **8** |
| --- | --- | --- | --- | --- | --- | --- | --- |
| Self-selected treadmill speed (km hr^-1^) | 2.3 | 2.3 | 1.9 | 1.3 | 1.6 | 4.8 | 2.9 |
| Treadmill incline (%) | 4 | 0 | 0 | 0 | 0 | 0 | 5 |
| Target HR GXT (bpm)^1^ | 82 | 88 | 80 | 104 | 121 | 110 | 127 |
| Target HR Age (bpm)^2^ | 92 | 81 | 87 | 108 | 121 | 106 | 123 |
|  |  |  |  |  |  |  |  |
| Average HR at F_I_O_2_ = 20.9% (bpm) | 84 | 82 | 81 | 112 | 145 | 121 | 118 |
| Average HR at F_I_O_2_ = 17.0% (bpm) | 84 | 88 | 81 | 112 | - | 132 | 120 |
| Average HR at F_I_O_2_ = 15.0% (bpm) | 89 | 90 | 81 | 116 | - | 144 | 120 |

^1^40 % of maximal HR recorded during graded exercise test; ^2^40 % of age predicted maximal HR; F_I_O_2_ = fraction of inspired oxygen
